# Mechanistic Insights into the Therapeutic Efficacy of Qi Ling Gui Fu Prescription in Broiler Ascites Syndrome: A Network Pharmacology and Experimental Study

**DOI:** 10.3390/vetsci12020078

**Published:** 2025-01-22

**Authors:** Jie Kang, Ruiqiang Deng, Keyao Wang, Huimin Wang, Yufeng Han, Zhibian Duan

**Affiliations:** 1 Science and Technology Center, Fenyang College of Shanxi Medical University, Lüliang 032200, China; kangjie219@163.com; 2 College of Veterinary Medicine, Shanxi Agricultural University, Jinzhong 030801, China; dengruiqiang@cnbizsg.com (R.D.); wangkeyao1206@163.com (K.W.); wanghuimin9805@163.com (H.W.); hanyufeng0904@163.com (Y.H.)

**Keywords:** network pharmacology, Qi Ling Gui Fu prescription, broiler ascites syndrome, MAPK pathway, phenotypic switching of VSMC

## Abstract

This study delves into the therapeutic potential of Qi Ling Gui Fu Prescription (QLGFP) in broiler ascites syndrome (AS) by investigating its impact on the phenotypic transformation of vascular smooth muscle. Utilizing network pharmacology, we identified 267 active ingredients and 120 core targets of QLGFP, revealing its multifaceted mechanism of action. Gene enrichment analysis highlighted the pivotal roles of Toll-like receptor, FoxO, and MAPK signaling pathways in QLGFP’s therapeutic effects. Experimental validation in a broiler AS model demonstrated that QLGFP regulated the expression of key markers (SM-22α, OPN, and KLF4) associated with the phenotypic transformation of pulmonary artery vascular smooth muscle (PASMC). Clinical improvements were evident, with a significant reduction in ascites cardiac index (AHI). Furthermore, QLGFP suppressed the protein expression of MAPK1 (ERK1), p-MAPK1, MAPK9 (JNK2), p-MAPK9, MA3.PK14 (P38α), and p-MAPK14, along with downstream factors AP1 and ATF4. These findings suggest that QLGFP effectively prevents and treats AS in broilers by modulating the MAPKs-AP1/ATF4 pathway, thereby inhibiting the phenotypic transformation and proliferation of PASMCs. This study contributes a theoretical foundation for understanding the role of QLGFP in the prevention and treatment of AS in broilers.

## Simple Summary

In this research, we explored the active components and therapeutic mechanisms of QLGFP for treating broiler AS using a network pharmacology approach. A total of 267 active compounds were isolated, of which the main active components were Dihydrokaranone, Tanshiquinone B, Neotanshinone C, etc. Bioinformatics analysis and an animal experiment indicated that the MAPK signalling pathway is the key target for QLGFP and their active compounds to improve the AS broilers by inhibiting phenotypic transformation of vascular smooth muscle. Our findings provide a scientific basis for the clinical efficacy of QLGFP in the treatment of broiler AS and a solid foundation for further elucidation of the active components and mechanism of action of QLGFP in the treatment of broiler AS.

## 1. Introduction

Broiler ascites syndrome (AS), also referred to as pulmonary hypertension syndrome (PHS), predominantly manifests in broilers aged 4–6 weeks. AS is characterized as a nutritional metabolic disease, presenting pathological changes in various organs, including right ventricular hypertrophy and dilation, pulmonary congestion and edema, and significant liver swelling and hydroabdomen [1]. Pulmonary hypertension (PH) is a major contributor to broiler AS [2]. Pulmonary vascular remodelling serves as a hallmark of PH, and the phenotypic change of pulmonary artery smooth muscle cells (PASMCs) forms the pathological basis for PH vascular remodelling. PASMCs exhibit two highly plastic phenotypes: ‘contraction’ and ‘synthesis’. Under normal circumstances, vascular smooth muscle cells (VSMCs) primarily have the ‘contraction phenotype’. Environmental changes induce VSMCs to transition from the ’contraction’ phenotype to the ’synthesis’ phenotype, marked by the downregulation of contraction marker protein (SM-22α) and the upregulation of synthesis or proliferation marker proteins (such as OPN, PCNA, and KLF4) [3,4,5,6]. This transition prompts migration to vascular injury sites and other lesions, fostering proliferation and promoting intimal hyperplasia. Recent studies underscore the pivotal role of the phenotypic transformation of PASMCs in PH vascular remodeling [7,8,9]. Drugs inhibiting the phenotypic transformation and proliferation of PASMCs hold promise for AS prevention and treatment.

Qi Ling Gui Fu Prescription (QLGFP) is an empirical formula developed in our laboratory for AS prevention and treatment. It comprises Chinese herbal compounds that invigorate qi, replenish and activate the blood, resolve stasis, induce diuresis, and remove dampness. QLGFP consists of seven herbal medicines (Astragalus Mongholicus, Poria, Angelica Sinensis, Salvia Przewalskii, Chuanxiong, licorice, and Areca Peel at a ratio of 3:2:2:2:2:1:1). Clinical experimental studies have demonstrated that QLGFP significantly improves clinical symptoms and organ functions in AS broilers. However, whether QLGFP can regulate the phenotypic transformation of PASMCs for AS prevention and treatment in broilers remains unstudied.

Traditional Chinese Medicine (TCM) is distinguished by its complex composition and broad range of targets. Chinese medicines act on various targets through diverse bioactive components, influencing multiple biological processes [10]. Network pharmacology, rooted in systems biology and multiple pharmacology theories, utilizes biomolecular networks to screen active compounds and potential therapeutic targets in Chinese medicine [11]. This approach offers a novel method to explore complex network relationships among multiple components and targets.

In this study, we employed network pharmacology to analyze the potential core components, key targets, and related molecular pathways of QLGFP in regulating the phenotypic transformation of PASMCs for AS prevention and treatment in broilers. The study elucidated the potential mechanism by which QLGFP prevents and treats AS in broilers through animal experiments.

## 2. Materials and Methods

### 2.1. Identification of Active Components and Targets of QLGFP

Astragalus Mongholicus, Poria, Angelica Sinensis, Salvia Przewalskii, Chuanxiong, liquorice, and Areca Peel were individually input into the Traditional Chinese Medicine Systems Pharmacology Database and Analysis Platform (TCMSP, https://old.tcmsp-e.com/index.php, accessed on 10 August 2022) using the criterion of ‘oral bioavailability (OB) ≥ 30%’ and ‘drug similarity (DL) ≥ 0.18’ to screen for active components and corresponding target proteins. Target gene names linked to the screened active ingredients were obtained through processing with the UniProt database (https://www.uniprot.org/, accessed on 10 August 2022). In addition, active compounds and targets of QLGFP were screened through the BATMAN-TCM database (http://bionet.ncpsb.org/batman-tcm/, accessed on 15 August 2022) with a score cut-off ≥ 20 and *p*-value cut-off < 0.05. Moreover, known targets of the active compounds were not cross-referenced with published literature. Combining all screening results yielded the pharmaceutically active ingredients and target genes of QLGFP.

### 2.2. Identification of Disease Targets

Utilizing the gene option of the NCBI and GeneCard (https://www.genecards.org, accessed on 20 August 2022) websites, disease-related gene targets were obtained by using search terms ‘ascites syndrome in broilers’, ‘Pulmonary Hypertension Syndrome in Broilers’, and ‘Phenotypic Transformation of Vascular Smooth Muscle’.

### 2.3. PPI Network Construction and Core Gene Screening

Disease-related targets and targets corresponding to the active components of QLGFP were input into Venn 2.1 software, and Venn diagrams were generated to identify common targets. The common target genes were analyzed using String (https://string-db.org, accessed on 3 September 2022) with a confidence level of 0.900 to construct a protein–protein interaction (PPI) file. Cytoscape with the CentiScape plug-in was used to calculate key values (intermediate centrality (BC), near centrality (CC), and activity score (DC)), and values greater than the average were screened to obtain a core gene PPI network.

### 2.4. GO and KEGG Pathway Enrichment Analysis

The core genes associated with the predicted drug–disease targets were analyzed through the DAVID database (https://david.ncifcrf.gov/, accessed on 12 September 2022) for both KEGG and GO enrichment. The significant pathways identified were then visualized using Cytoscape to create a network map. A bubble chart representing these key pathways was generated using the Bioinformatics platform (http://www.bioinformatics.com.cn/, accessed on 15 September 2022).

### 2.5. Preparation of QLGFP

Traditional Chinese medicines, including Astragalus Mongholicus, Poria, Angelica Sinensis, Salvia Przewalskii, Chuanxiong, liquorice, and Areca Peel, were formulated in a ratio of 3:2:2:2:2:1, soaked in clean water for 30 min, decocted for 40 min, filtered with gauze, and collected as liquid medicine. This process was repeated three times, and the combined liquid medicine was distilled and concentrated to 1 g/mL, stored at −20 °C for later use, and warmed before feeding.

### 2.6. Establishment of the Broiler Ascites Syndrome Model and Grouping

Eighty-seven day-old healthy Ross broilers were randomly divided into four groups: normal (N), model (M), and QLGFP high- and low-dose groups (H, L). The model involved raising broilers at a low temperature (9–11 °C) from the age of 8 days, with the basic diet supplemented with high-fat (3% lard), high-protein (4% fish meal), and high-sodium (0.12% NaCL) drinking water. The high- and low-dose groups were given 2.0 and 1.0 g/kg compound Chinese medicinal decoctions per day, respectively.

### 2.7. Sample Collection and Preparation

Ten broilers were randomly selected from each group at the age of 35 days. Hearts were dissected for determining the ascites cardiac index (AHI). AHI, which refers to the ratio of right ventricular weight to total ventricular weight (RV/TV), is used to diagnose ascites in broilers, with a value above 0.250 being considered indicative of the condition [12,13]. Additionally, a segment of the pulmonary artery was collected, quickly frozen in liquid nitrogen, and stored at −80 °C for subsequent protein and gene detection. The remaining portion of the pulmonary artery was preserved in 10% formaldehyde for histopathological examination.

### 2.8. Pulmonary Artery Histopathological Observation

Using the conventional paraffin section method, sections with a thickness of approximately 5 μm were cut and stained with hematoxylin and eosin (HE) staining for observing structural changes and Masson staining for observing collagen fiber deposition in the pulmonary artery tissue.

### 2.9. Immunofluorescence

Paraffin sections were dewaxed, underwent microwave antigen repair, blocking with 5% bovine serum albumin, and incubation with diluted primary antibody PCNA (1:100) at 37 °C for 2 h. Following overnight refrigeration at 4 °C, secondary antibody (488-labeled goat anti-mouse IgG) was added, and after incubation, washing, dehydration, and sealing, observations were made under a fluorescence microscope.

### 2.10. Detection of Protein Content by ELISA

The pulmonary artery tissue was slowly rinsed in ice-cold normal saline, washed, water-absorbed, weighed, mixed with normal saline at a ratio of 1:9 (*w*/*v*), and homogenized. The sample was then centrifuged at 3000 rpm for 20 min to collect the supernatant. Protein levels were determined using an ELISA kit according to the manufacturer’s guidelines.

## 3. Results

### 3.1. Active Ingredient–Disease Intersection Target Network

**Figure 1:**
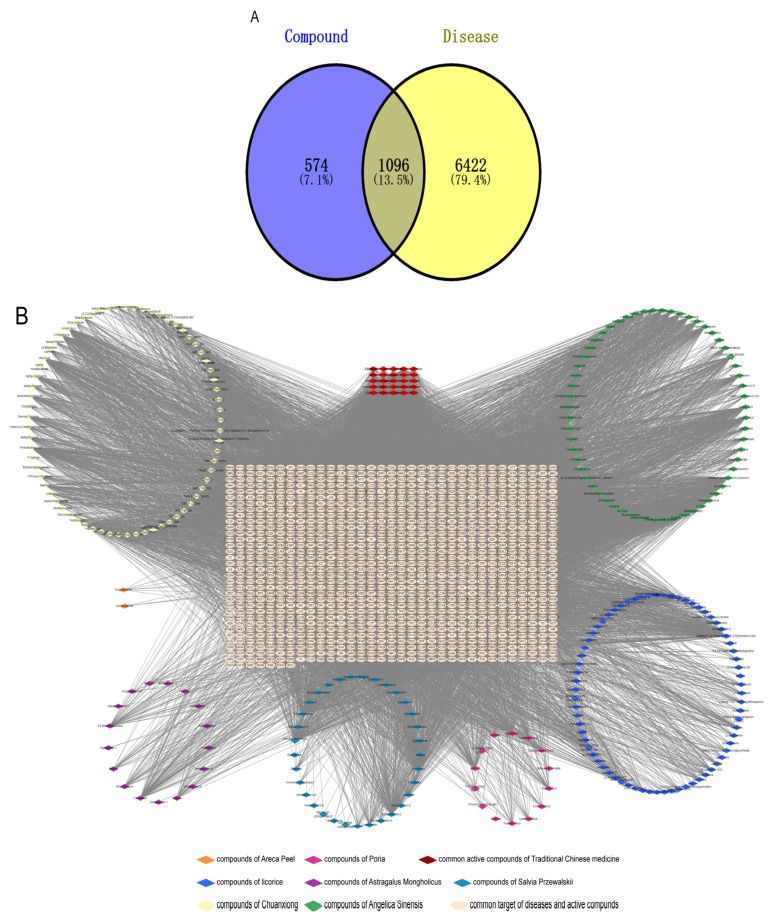
Active ingredient–disease intersection target network. (**A**): Venn diagram; (**B**): the single herb–active ingredient–disease target intersection network.

Utilizing the TCMSP and BATMAN-TCM databases, we identified 267 active components of QLGFP (Table S1), with specific counts for each herb: seventeen components from Astragalus Mongholicus, fourteen components from Poria, sixty-one components from Angelica Sinensis, thirty-three components from Salvia Przewalskii, sixty-nine components from Chuanxiong, seventy-one components from liquorice, and two components from Areca Peel. Twenty-five active ingredients were found to be common to two or more herbs. In addition, we identified 1670 potential targets corresponding to these active compounds. The disease-related targets for pulmonary hypertension syndrome and phenotypic transformation of vascular smooth muscle in broilers were gathered from the NCBI and GeneCard databases, resulting in 7518 targets. Venn diagram analysis revealed 1096 intersecting targets shared by both QLGFP and the disease (Figure 1A).

The network relationship between the intersection targets of active components in the single herb–active ingredient–disease target was constructed using Cytoscape software (Figure 1B). In addition, of the 267 bioactive components, the top 15 core components, deemed most effective against AS, such as Dihydrokaranone, Tanshiquinone B, and Neotanshinone C, were identified (Table 1).

### 3.2. Construction of the PPI Network and Screening of the Core Genes

**Figure 2:**
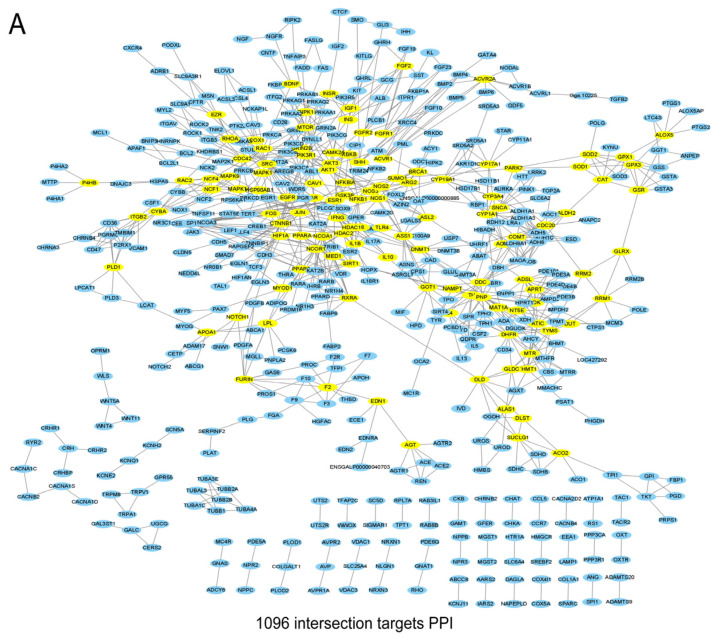

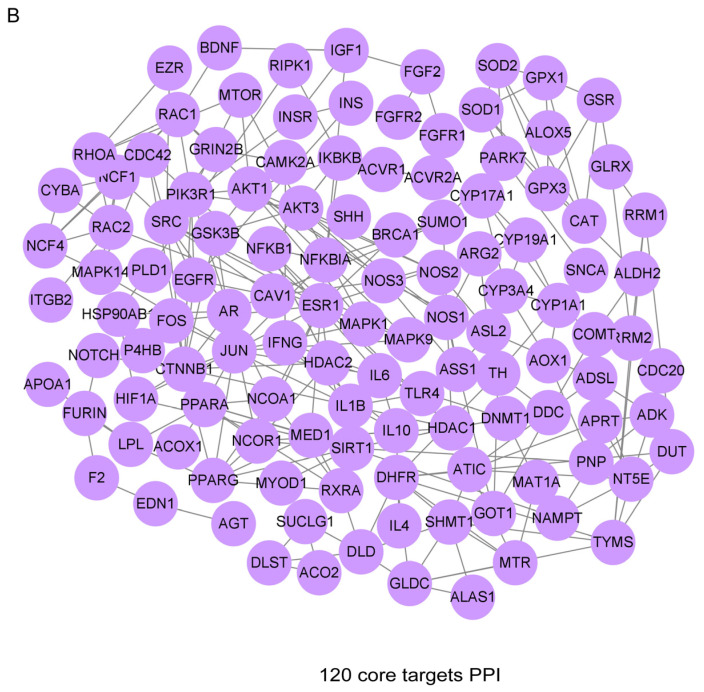
PPI protein interaction network. protein–protein interaction network. (**A**): PPI network for 1096 intersecting targets (**B**): 120 core targets identified by CentiScape.

The 1096 intersecting targets were uploaded to the String database, where a PPI network was created with a confidence threshold of 0.9 (Figure 2A). The CentiScape plug-in was used to identify 120 core targets based on metrics such as DC, BC, and CC. Core genes included Jun, PIK3R1, FOS, MAP K14, NFKB1, IKbKB, NFKBIA, MAP K9, IL6, IL1b, AKT3, AKT1, RIPK1, RAC1, TLR4, and MAPK1 (Figure 2B).

### 3.3. Functional Enrichment Analysis

**Figure 3:**
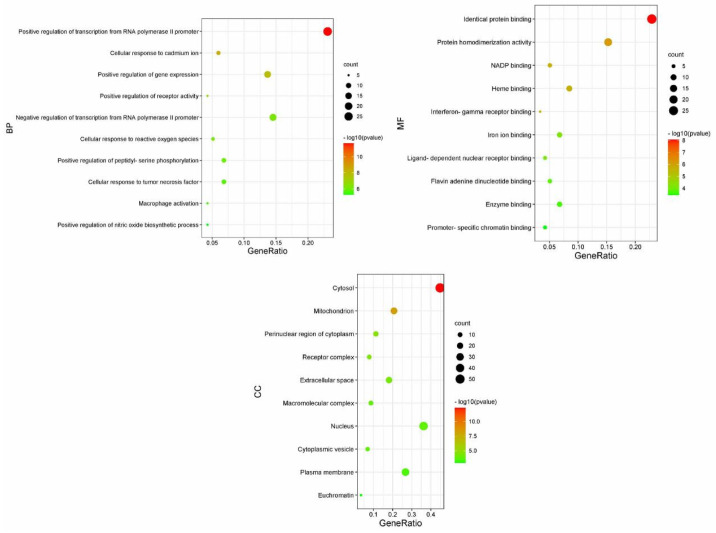
Bubble diagram for GO functional enrichment analysis. GO, Gene Ontology.

**Figure 4:**
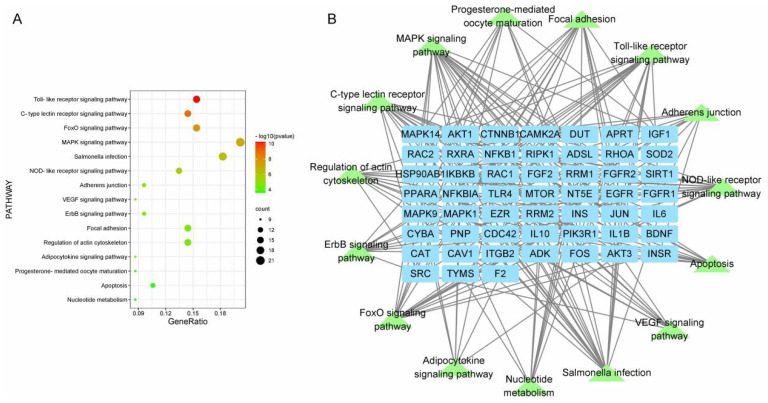
(**A**): Bubble diagram for KEGG pathway enrichment analysis; (**B**): pathway–core target network diagram. KEGG, Kyoto Encyclopedia of Genes and Genomes.

GO functional enrichment of the 120 core targets identified 345 biological process (BP) terms; these primarily involved the enhancement of transcription from the RNA polymerase II promoter, cellular response to cadmium ions, activation of gene expression, and stimulation of receptor activity. They also included the suppression of transcription from the RNA polymerase II promoter and the cellular response to reactive oxygen species (ROS), positive regulation of peptidyl-serine phosphorylation, cellular [14] response to tumor necrosis factor, macrophage activation, and positive regulation of the nitric oxide biosynthetic process, among others. A total of 78 enriched terms were identified in the molecular function (MF) category, primarily associated with identical protein binding and protein homodimerization activity, NADP binding, heme binding, interferon-gamma receptor binding, iron ion binding, ligand-dependent nuclear receptor, flavin adenine dinucleotide, enzyme, and promoter-specific chromatin binding. Additionally, 44 terms were found in the cellular component (CC) category, which were ranked based on the adjusted *p*-value. The top 10 terms were chosen for visualization in a bubble chart (Figure 3). Furthermore, KEGG enrichment analysis identified 47 signaling pathway terms. These included signaling pathways such as the Toll-like receptor, C-type lectin receptor, and FoxO, MAPK, salmonella infection, NOD-like receptor, adheren junction, VEGF, ErbB signaling pathway. They also encompassed focal adhesion, regulation of the actin cytoskeleton, adipocytokine signaling pathway, progesterone-mediated oocyte maturation, apoptosis, and nucleotide metabolism. The first 15 pathways were selected for the bubble chart display, as shown in Figure 4A. A pathway–core target network diagram was constructed, as shown in Figure 4B. The targets of the main active components of QLGFP were distributed across different pathways, such as Toll-like receptor signaling pathway, FoxO signaling pathway, MAPK signaling pathway with related genes. The KEGG and pathway–core target network analysis indicated that multi-component, multi-target, and multi-pathway mutual regulation are possible mechanisms for the prevention and treatment of AS in broiler. 

### 3.4. Effect of QLGFP on the Ascites Heart Index of Broilers

**Figure 5:**
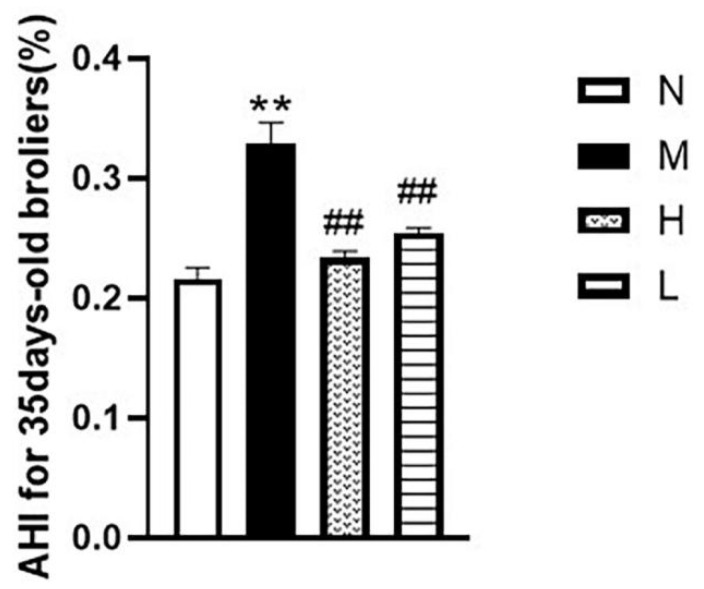
Effects of QLGFP on the AHI of 35-day-old AS broilers (*n* = 3). ** *p* < 0.01, vs. normal group; ## *p* < 0.01 vs. model group.

As shown in Figure 5, the AHI results demonstrated a significant reduction in the QLGFP high- and low-dose groups compared to the model group, indicating the potential efficacy of QLGFP in mitigating AS (*p* < 0.01).

### 3.5. The Influence of QLGFP on Pulmonary Artery Pathology

**Figure 6:**
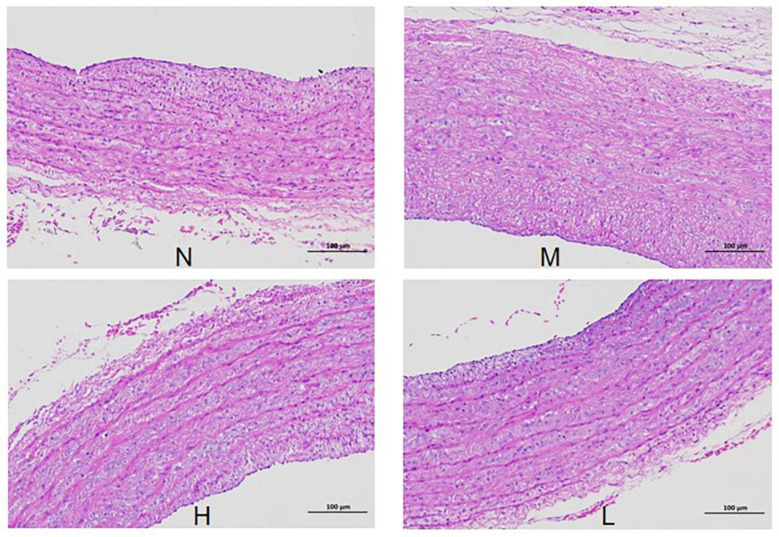
Effects of QLGFP on pathological changes in the pulmonary artery of 35-day-old broilers (HE staining, 100×); HE, hematoxylin and eosin.

**Figure 7:**
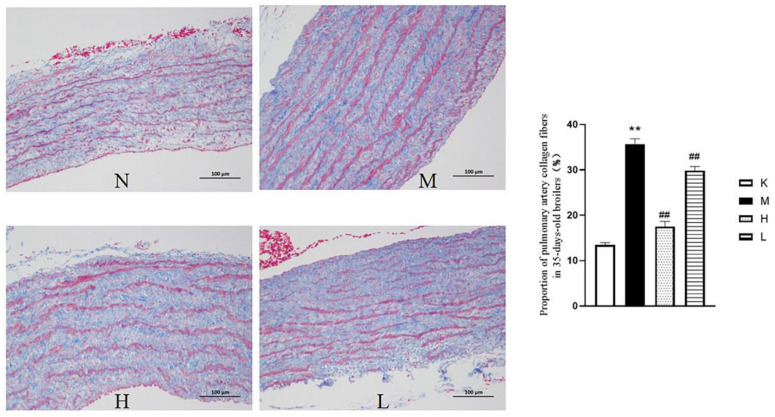
Effects of QLGFP on pathological changes in the pulmonary arteries and the proportion of pulmonary collagen fibers in 35-day-old broilers (Masson staging, 100×). ** *p* < 0.01, vs. normal group; ## *p* < 0.01, vs. model group.

As shown in Figure 6, the HE staining results showed that compared with the normal group (N), the membrane in the vascular smooth muscle of the model group (M) was thickened and the cell volume was increased. Compared with the model group (M), the high and low doses of QLGFP in the H and L groups could significantly reduce these phenomena. As shown in Figure 7, the Masson staining results show that, compared with the normal group (N), the collagen fiber content in the pulmonary artery tissue of the model group (M) was significantly increased (*p* < 0.01). Compared with the model group (M), the collagen fiber contents were significantly reduced in both the H and L groups (*p* < 0.01).

### 3.6. Observation of PCNA Expression in the Pulmonary Artery by Immunofluorescence and Marker Proteins

**Figure 8:**
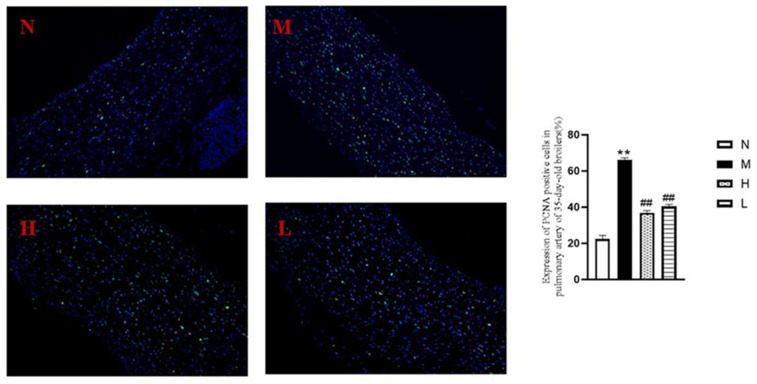
Immunofluorescence to detect the expression of PCNA-positive cells in pulmonary arteries (200×) and the proportion of positive expression (*n* = 3). ** *p* < 0.01, vs. normal group; ## *p* < 0.01, vs. model group.

**Figure 9:**
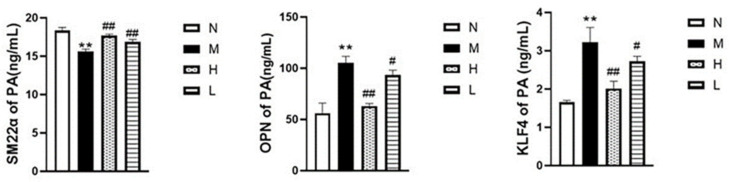
Effects of QLGFP on marker expression levels in the pulmonary arteries of 35-day-old broilers. (*n* = 3). ** *p* < 0.01, vs. normal group; ## *p* < 0.01, # *p* < 0.05 vs. model group.

As illustrated in Figure 8, the model group (M) showed a significantly higher number of PCNA-positive cells in the pulmonary artery tissue compared to the normal group (N) (*p* < 0.01). In contrast, both the H and L groups exhibited a notable reduction in PCNA-positive cell expression compared to the model group (M) (*p* < 0.01). Additionally, ELISA results demonstrated a positive effect on marker proteins related to the phenotypic transformation of pulmonary artery smooth muscle cells (Figure 9). In the model group (M), the contraction marker SM22α was significantly downregulated (*p* < 0.01), while the synthetic markers OPN and KLF4 were upregulated (*p* < 0.01), compared to the normal group (N). However, in the H and L groups, SM22α expression increased, while OPN and KLF4 levels decreased significantly compared to the model group (M) (*p* < 0.01). (*p* < 0.05).

### 3.7. Effects of QLGFP on the MAPK Pathway

**Figure 10:**
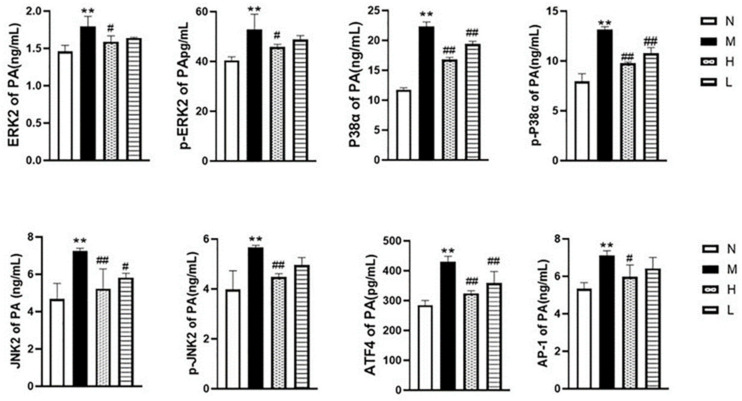
Effects of QLGFP on the protein content of MAPK pathway-related factors in the pulmonary artery of 35-day-old broilers (*n* = 3). ** *p* < 0.01, vs. normal group; ## *p* < 0.01, # *p* < 0.05 vs. model group.

According to the ELISA results (Figure 10), compared to the normal group (N), the expression of MAPK1 (ERK1), p-MAPK1, MAPK9 (JNK2), p-MAPK9, MAPK14 (P38α), p-MAPK14, AP1, and ATF4 in the model group (M) increased (*p* < 0.01). Compared with the model group (M), the expression of MAPKs, AP1, and ATF4, as well as the phosphorylation of MAPK proteins, was significantly inhibited in the high-dose QLGFP group (H) (*p* < 0.01) (*p* < 0.05). The low-dose QLGFP group (L) showed inhibition, which was weaker than the high-dose group (H). These findings collectively suggest that QLGFP may exert preventive and therapeutic effects on Broiler Ascites Syndrome by influencing multiple targets and pathways associated with the phenotypic transformation of pulmonary artery smooth muscle cells.

## 4. Discussion

The traits of modern broiler breeds, such as high metabolic activity, rapid growth, and increased meat production, have led to a notable rise in the occurrence and mortality rates associated with broiler ascites syndrome (AS) [1,15,16] To sustain optimal broiler performance, developing natural medications or novel feed additives to alleviate AS is crucial. In recent years, TCM has gained global acceptance for its multi-target and multi-level functional effects [17]. Therefore, we investigated the therapeutic mechanism of QLGFP in treating AS in broilers, a study of paramount significance for AS prevention and treatment.

This study takes into account the complexity of QLGFP compounds and the range of potential targets. We initiated a screening process for compound targets in QLGFP and broiler AS disease-related targets, utilizing network pharmacology across multiple databases. A target network of QLGFP single-herb compounds was constructed, predicting main active components such as Dihydrokaranone, Tanshiquinone B, and Neotanshinone C. Saturated fatty acids (FAs) such as hexadecanoic acid (palmitic acid) and linoleic acid can play important roles in poultry growth, such as energy supply, normal cell structure and function, and maintaining immune system regulation [18]. Tanshiquinone B and Neotanshinone C. showed a high affinity with proinflammatory cytokines such as TNF-α, interleukin 1 beta (IL-1β), and interleukin 6 (IL-6) to suppress inflammatory response [19]. Furthermore, the core targets of QLGFP in broiler AS treatment were identified. Through PPI network analysis, our research found that the key active components of QLJP play a role in regulating cell proliferation, migration, and apoptosis, antioxidant stress, anti-inflammation, and other biological effects. Broiler hypoxia triggers AS, resulting in tissue and cell damage, excessive cell proliferation, and anti-apoptosis [20,21], and subsequent pulmonary dysfunction, vascular smooth muscle phenotypic transformation, pulmonary vascular remodelling, and PAH. Our findings suggested that QLGFP interferes with the molecular mechanism of AS in broilers by regulating oxidative stress, inflammatory responses, cell proliferation, migration, and phenotypic transformation.

Previous studies have reported organ damage in broilers due to enhanced performance, along with a marked increase in AHI. Dynamic fluctuations in AHI indicate the progression of AS, with lung injury potentially serving as a trigger for the condition [22]. Similarly, our study revealed a substantial increase in the AHI values in the model group. PH stands as a pivotal link in AS pathogenesis, primarily arising from pulmonary artery lacunar stenosis due to pulmonary vascular remodelling. The transformation of VSMC from a differentiated phenotype with contractile function to a synthetic phenotype with strong proliferation, migration, and secretion capabilities plays an important role in pulmonary vascular remodelling. Therefore, inhibiting the phenotypic transformation of VSMC is an important strategy for AS prevention and treatment.

Histopathological analyses, including HE and Masson staining, unveiled thickening of the membrane and increased cell volume in the vascular smooth muscle of the model group, accompanied by a notable rise in collagen fiber content in pulmonary artery tissue. However, membrane thickening and tissue fibrosis exhibited marked improvement in the treatment group. Immunofluorescence results further demonstrated the significant inhibition of VSMC proliferation with drug treatment.

SM22α is involved in cytoskeletal remodelling and phenotypic modulation of VSMCs by interacting with actin, and regulates the activity of signal transduction molecules related to cell proliferation, oxidative stress, and inflammation [23,24]. The combination of KLF4 with G/C elements inhibits the expression of the SM22α gene [25]. OPN is involved in a variety of physiological and pathological processes and has chemotactic and cell proliferation-promoting effects. Studies have shown that SM22α and OPN are closely related to the biological function of VSMCs [26,27]. Our ELISA results showed an upregulation of contractile marker gene expression and a decrease in synthetic marker gene expression (OPN and KLF4) compared to the model group, signifying that the compounds could ameliorate AS by inhibiting the phenotypic transformation of VSMC.

Furthermore, KEGG analysis suggested that the core targets of QLGFP may be associated to Toll-like receptor, FoxO, and MAPK signalling pathways. The MAPK signalling cascade, recognized for its pivotal role in the pathogenesis of cardiovascular diseases, was found to be implicated in the pathogenesis of AS. Activation of MAPKs, extracellular signal-regulated kinase (ERK), c-Jun N-terminal kinase (JNK), and p38 MAPK is closely tied to the phenotypic transformation of vascular smooth muscle, atherosclerosis, and cardiac remodelling after vascular restenosis [28]. Our results indicated that QLGFP could inhibit the MAPK signalling pathway, thereby effectively suppressing the phenotypic transformation and proliferation of vascular smooth muscle, ultimately reducing PAH and impeding AS progression. Hu et al. [29] found that icariin inhibits the abnormal proliferation of smooth muscle cells by blocking the p38MAPK signalling pathway and reducing the expression of proliferating cell nuclear antigen (PCNA). Further, Tong et al. [14] found that Shijumuli Decoction could reduce the blood pressure of spontaneously hypertensive rats, reverse aortic vascular remodelling, and inhibit the expression of p-p38MAPK, which might be related to its inhibition of the activation of the p38MAPK signalling pathway, thereby reducing the damage to endothelial cells and inhibiting phenotypic transformation, proliferation, and migration of smooth muscle cells. Recent studies have shown that arterial stretch caused by the physicochemical factors of hypertension can promote the phosphorylation of ERK1/2/P38/JNK MAPK in VSMCs to increase their proliferation, migration, and apoptosis as well as promote arteriosclerosis [30], and cortistatin showed the potential in antiproliferation and antimigration effects in vascular smooth muscle cells stimulated by Ang II through suppressing ERK1/2/p38 MAPK/JNK signaling pathways [31]. The increase in angiotensin II activity caused by hypertension can activate MAPK (P38/JNK/ERK) expression to promote excessive VSMC proliferation and oxidative stress [32].

Su et al. [33] investigated the mechanism through which ROS regulate VSMC differentiation. They discovered that ROS could induce the expression of phenotypic marker proteins associated with VSMC differentiation, thereby promoting the phenotypic transformation of VSMCs by activating the p38MAPK pathway. AP-1 and ATF4, transcription regulatory proteins, serve as conduits for transmitting extracellular stimulation signals to the nucleus, initiating the transcription of target genes at their respective sites. These proteins play an important role in cell proliferation, differentiation, transformation, apoptosis, and extracellular matrix accumulation, acting as the primary molecular regulator in the transformation of VSMCs into a hyperplastic phenotype [34].

Studies have shown that the increased bindings of AP-1 to the OPN promoter lead to increased OPN expression, thereby inducing VSMC proliferation and subsequent vascular remodelling. Additionally, investigations into the transcriptional induction of the SM22α gene by TGF-β have revealed the contributory role of AP-1 elements in the induction of the SM22α promoter by TGF-β [35]. Recent study exploring the role of human erythropoietin in the proliferation, migration, and invasion of VSMC indicated that the p38MAPK signaling pathway is implicated in VSMC migration and invasion in response to the EPO gene, facilitated by the activation of the AP-1 binding motif [35]. Similarly, the p38 MAPK/CREB signaling pathway has been identified in the expression of phenotypic marker proteins associated with VSMC differentiation, as observed in the regulatory effects of Cyanin-3-O-β-glucoside on cell proliferation [36].

A comprehensive network pharmacology analysis highlighted the MAPKs-AP1/ATF4 pathway as the principal target of these compounds. Subsequent animal experiments demonstrated that the compound Chinese medicine inhibited the expression of MAPK pathway-related factors, such AS MAPK1, MAPK9, MAPK14, P-MAPK1, P-MAPK9, and P-MAPK14, along with downstream pathway factors, such as AP1 and ATF4. Consequently, we speculate that compound Chinese medicines may inhibit the phenotypic transformation of vascular smooth muscle by modulating the expression of transformation-related proteins through the MAPKs-AP1/ATF4 pathway, thereby effectively thwarting the onset and progression of AS.

## 5. Conclusions

This study explored the active ingredients and underlying mechanisms of QLGFP in treating broiler AS through a network pharmacology approach. A total of 267 active compounds were isolated, of which the main active components were Dihydrokaranone, Tanshiquinone B, Neotanshinone C, etc. Through KEGG enrichment analysis, 47 signal transduction pathways of QLGFP in AS broilers were revealed, and the key mechanisms were related to the Toll-like receptor signalling pathway, FoxO signalling pathway, and MAPK signalling pathway. In the animal experiments, the clinical and anatomical signs observed in the model group of broilers aligned with the typical characteristics of AS in broilers as reported. The AHI values significantly increased. We determined the protective effects and potential mechanisms of action of QLGFP in AS broilers. QLGFP may inhibit the MAPK signalling pathway, inhibit phenotypic transformation and proliferation of vascular smooth muscle, and reduce PAH, thereby reducing the occurrence and development of AS. Our results offer scientific support for the clinical effectiveness of QLGFP in treating broiler AS and lay a strong foundation for further investigation into the active components and mechanisms involved in its therapeutic action.

## Figures and Tables

**Table 1:** Top 15 active compounds.

PubChem Cid	Molecule Name	Formula	CAS Number	Structure	Target Amount
177072	Dihydrokaranone	C_15_H_22_O	19598-45-9	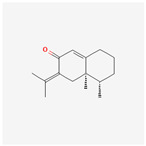	203
5319799	3-Methyl-6,7,8-Trihydropyrrolo [1,2-A]Pyrimidin-2-One	C_8_H_10_N_2_O	76884-47-4	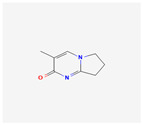	199
5320113	Tanshiquinone B	C_18_H_16_O_3_	65907-76-8	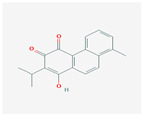	168
5320114	Neotanshinone C	C_16_H_12_O_3_	65907-77-9	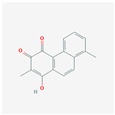	168
5320066	Neocryptotanshinone Ii	C_17_H_18_O_3_	27468-20-8	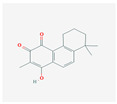	168
5319835	Miltionone I	C_19_H_20_O_4_	6855-99-8	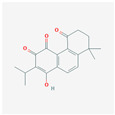	168
102090422	Sebiferic Acid	C_30_H_48_O_2_	52809-09-3	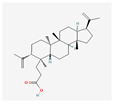	154
5417	Tetrahydropalmatine	C_21_H_25_NO_4_	2934-97-6	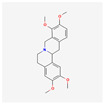	138
13849	Pentadecanoic Acid	C_15_H_30_O_2_	1002-84-2	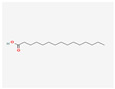	138
23518	Methyl Pentadecanoate	C_16_H_32_O_2_	7132-64-1	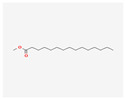	138
985	Hexadecanoic Acid	C_16_H_32_O_2_	67701-02-4	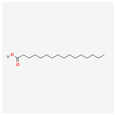	138
2969	Decanoic Acid	C_10_H_20_O_2_	334-48-5	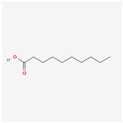	138
19347555	Azelaic Acid	C_9_H_16_O_4_	32733-99-6	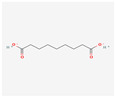	138
12113497	13-Methyl Pentadecanoic Acid	C_16_H_32_O_2_	20121-96-4	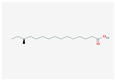	138
5280450	Linoleic Acid	C_18_H_32_O_2_	2197-37-7	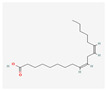	136

## Data Availability

All data are available.

## Supplementary Materials

The following supporting information can be downloaded at: https://www.mdpi.com/article/10.3390/vetsci12020078/s1. Table S1: 267 active compounds screened by Network Pharmacology.

## Abbreviations

The following abbreviations are used in this manuscript:

QLGFP	Qi Ling Gui Fu Prescription
AS	broiler ascites syndrome
PH	Pulmonary hypertension
PASMC	Linear dichroism pulmonary artery smooth muscle cells
TCM	Traditional Chinese Medicine
OB	oral bioavailability
KEGG	Encyclopedia of Genes and Genomes
GO	Gene Ontology
HE	Hematoxylin and eosin
ELISA	Enzyme-linked immunosorbent assay
