# Supplementary material for: Mechanistic Insights into the Therapeutic Efficacy of Qi Ling Gui Fu Prescription in Broiler Ascites Syndrome: A Network Pharmacology and Experimental Study
Source: Vet Sci. 2025 Jan 22;12(2):78. doi: 10.3390/vetsci12020078 (PMC11860255; doi:10.3390/vetsci12020078)
Supplement: Supplementary file 1 [file vetsci-12-00078-s001.zip › vetsci-3418618-supplementary.pdf]

Table S1 267 active compounds screened by Network Pharmacology

S/N	Compound	PubChem CID	Herb
1	Astragaloside Vi	71448940	Astragalus Mongholicus
2	Astramembrannin I	146159870	Astragalus Mongholicus
3	Beta-Sitosterol	222284	Astragalus Mongholicus
4	Betaine	247	Astragalus Mongholicus
5	Soyasapogenol B	115012	Astragalus Mongholicus
6	Kaempferol	5280863	Astragalus Mongholicus
7	Quercetin	5280343	Astragalus Mongholicus
8	Astragaloside V	71448939	Astragalus Mongholicus
9	Astragaloside Iii	441905	Astragalus Mongholicus
10	Astragaloside I	13996685	Astragalus Mongholicus
11	3,5-Dimethoxystilbene	5316874	Astragalus Mongholicus
12	Sucrose	5988	Astragalus Mongholicus
13	Canavanine	439202	Astragalus Mongholicus
14	Astragaloside Vii	14241100	Astragalus Mongholicus
15	Astragaloside Ii	13996693	Astragalus Mongholicus
16	Soyasaponin 1	122097	Astragalus Mongholicus
17	Lupeol	259846	Astragalus Mongholicus
18	Hydrangeic Acid	5318116	Poria
19	Eburicol	9803310	Poria
20	O-Acetylpachymic Acid-25-Ol	5316009	Poria
21	Poricoic Acid B	3086648	Poria
22	Ergotamine	8223	Poria
23	Undecan-2-Ol	15448	Poria
24	Turanose	5460935	Poria
25	Lauric Aldehyde	329758109	Poria
26	Ergosterol	444679	Poria
27	Dehydroeburicoicacid	15250826	Poria
28	Pachymic Acid	5484385	Poria
29	Beta-Amyrin Acetate	92156	Poria
30	P-Hydroxybenzyl Alcohol	125	Poria
31	10-Hydroxyacetylbaccatin Vi	53145052	Poria
32	Dimethyl Phthalate	8554	Angelica Sinensis
33	2-Methyl-Dodecane-5-One	5319599	Angelica Sinensis
34	Hexadecanoic Acid	985	Angelica Sinensis
35	Scopolin	439514	Angelica Sinensis
36	Dimethyl Azelate	15612	Angelica Sinensis
37	Dodecenoic Acid	12736	Angelica Sinensis
38	Dimethyl-Beta-Propiothetin	23736	Angelica Sinensis
39	4-Ethylresorcinol	17927	Angelica Sinensis
40	Beta-Myrcene	31253	Angelica Sinensis
41	Phenylacetic Acid	999	Angelica Sinensis
42	1-Tridecene	17095	Angelica Sinensis

43	Sebiferic Acid	5321206	Angelica Sinensis
44	Scopoletin	5280460	Angelica Sinensis
45	Angelicin	10658	Angelica Sinensis
46	Ethyl-P-Methoxycinnamate	3032313	Angelica Sinensis
47	Decanoic Acid	2969	Angelica Sinensis
48	12-O-Nicotinoylisolineolone	5320138	Angelica Sinensis
49	Dihydropinosylvin	442700	Angelica Sinensis
50	Ethanol	702	Angelica Sinensis
51	Angelicide	5316848	Angelica Sinensis
52	Bicycloelemene	56842786	Angelica Sinensis
53	Alloocimene	5368821	Angelica Sinensis
54	1-Methyl-2-Dodecyl-4-(1h)-Quinolone	5319601	Angelica Sinensis
55	Beta-Bisabolene	10104370	Angelica Sinensis
56	6-Undecanol	32045	Angelica Sinensis
57	M-Ethylphenol	12101	Angelica Sinensis
58	Carvacrol Acetate	80792	Angelica Sinensis
59	Alpha-Acoradiene	6429151	Angelica Sinensis
60	Isococculidine	442300	Angelica Sinensis
61	1,2-Benzenedicarboxylic Acid	1017	Angelica Sinensis
62	Dimethyl Camphorate	348846	Angelica Sinensis
63	Carvacrol	10364	Angelica Sinensis
64	Cedrol	65575	Angelica Sinensis
65	1-Tetradecanol	8209	Angelica Sinensis
66	Anisic Acid	7478	Angelica Sinensis
67	Phyllanthin	358901	Angelica Sinensis
68	P-Cresol	2879	Angelica Sinensis
69	2',4'-Dihydroxyacetophenone	6990	Angelica Sinensis
70	2-Methyl-3-Buten-2-Ol	8257	Angelica Sinensis
71	1,2-Dimethylbenzene	7237	Angelica Sinensis
72	Beta-Acoradiene	20055537	Angelica Sinensis
73	Decanal	8175	Angelica Sinensis
74	Brefeldin A	5287620	Angelica Sinensis
75	Stigmasterol	5280794	Angelica Sinensis
76	Nonanal	31289	Angelica Sinensis
77	Vitamin B12	5311498	Angelica Sinensis
78	1-Hexadecanol	2682	Angelica Sinensis
79	4-Octanone	11516	Angelica Sinensis
80	P-Ethylphenol	31242	Angelica Sinensis
81	Campherenol	91747494	Angelica Sinensis
82	Beta-Caryophyllene	5281515	Angelica Sinensis
83	1-Dodecene	8183	Angelica Sinensis
84	Guaiacol	460	Angelica Sinensis
85	Isoeugenol	853433	Angelica Sinensis
86	M-Cresol	342	Angelica Sinensis

87	Isofernene	5318564	Angelica Sinensis
88	Hexanoic Acid	8892	Angelica Sinensis
89	Crinamine	73620	Angelica Sinensis
90	6-Undecanone	13561	Angelica Sinensis
91	Alpha-Chamigrene	442351	Angelica Sinensis
92	Azelaic Acid	2266	Angelica Sinensis
93	Tanshiquinone B	5320113	Salvia Przewalskii
94	Danshenxinkun A	149138	Salvia Przewalskii
95	Dehydromiltirone	3082765	Salvia Przewalskii
96	Ferruginol	442027	Salvia Przewalskii
97	Neocryptotanshinone	389888	Salvia Przewalskii
98	Danshensu	11600642	Salvia Przewalskii
99	Neocryptotanshinone Ii	15690458	Salvia Przewalskii
100	Heteratisine	431671	Salvia Przewalskii
101	Isotenulin	442265	Salvia Przewalskii
102	Miltirone	160142	Salvia Przewalskii
103	Tanshinone Vi	49138	Salvia Przewalskii
104	Salvianolic acid B	6451084	Salvia Przewalskii
105	Daphneolone	5316300	Salvia Przewalskii
106	Dehydrotremetone	324281	Salvia Przewalskii
107	Lithospermate B	6438135	Salvia Przewalskii
108	Dauricine	73400	Salvia Przewalskii
109	Rosmarinine	5281745	Salvia Przewalskii
110	Isocucurbitacin D	6325422	Salvia Przewalskii
111	6-Hydroxymethyllumazin	6325373	Salvia Przewalskii
112	Tanshinone Iib	9926694	Salvia Przewalskii
113	Samaderin A	441807	Salvia Przewalskii
114	1-Hydroxytaxinine A	5315909	Salvia Przewalskii
115	Ursolicacid	45358157	Salvia Przewalskii
116	Dihydrokaranone	10353347	Salvia Przewalskii
117	Salviol	13966146	Salvia Przewalskii
118	Magnesium Lithospermate B	6918234	Salvia Przewalskii
119	Miltionone I	5319835	Salvia Przewalskii
120	Monomethyl Lithospermate	95224420	Salvia Przewalskii
121	Salonitenolide	5281498	Salvia Przewalskii
122	Lithospermic Acid	6441498	Salvia Przewalskii
123	Neotanshinone C	5320114	Salvia Przewalskii
124	Salvinone	130233	Salvia Przewalskii
125	Cryptoxanthin	5281235	Salvia Przewalskii
126	Gamma-Terpinene	7461	Chuanxiong
127	L-Valine-L-Valine Anhydride	5315167	Chuanxiong
128	P-Cymene	7463	Chuanxiong
129	Fenchone	14525	Chuanxiong
130	Pentadecanol	12397	Chuanxiong

131	Methyl Phenyl Carbinol	7409	Chuanxiong
132	Cnidium Lactone	5315981	Chuanxiong
133	Hexadecanol	2682	Chuanxiong
134	Beta-Eudesmol	91457	Chuanxiong
135	Enanthaldehyde	8130	Chuanxiong
136	Borneol	64685	Chuanxiong
137	Heptadecanol	15076	Chuanxiong
138	Dibutyl Phthalate	3026	Chuanxiong
139	Spathulenol	92231	Chuanxiong
140	Terpinen-4-Ol	11230	Chuanxiong
141	Beta-Pinene	14896	Chuanxiong
142	Octanol	957	Chuanxiong
143	Phenylacetaldehyde	998	Chuanxiong
144	Camphor	2537	Chuanxiong
145	Citronellol	8842	Chuanxiong
146	Chrysophanol	10208	Chuanxiong
147	Trans-Caryophyllene	5281515	Chuanxiong
148	Linalool	6549	Chuanxiong
149	Myrcene	31253	Chuanxiong
150	Cibarian	100275	Chuanxiong
151	(Z,Z')-Diligustilide	70698035	Chuanxiong
152	Thymol	6989	Chuanxiong
153	Methyl Eugenol	7127	Chuanxiong
154	Menthyl Acetate	27867	Chuanxiong
155	Vallesiachotamine	5384527	Chuanxiong
156	5,6-Dihydroergosterol	13889661	Chuanxiong
157	Ethyl Linoleate	5282184	Chuanxiong
158	Methyl Palmitate	8181	Chuanxiong
159	13-Methyl Pentadecanoic Acid	146501	Chuanxiong
160	Alpha-Phellandrene	7460	Chuanxiong
161	Caffeic Acid Dimethyl Ether	717531	Chuanxiong
162	Oleic Acid	445639	Chuanxiong
163	1-Methoxy-2-Methylanthraquinone	3534338	Chuanxiong
164	Senkyunolide G	10013283	Chuanxiong
165	24-Ethylidene Lophenol	101614269	Chuanxiong
166	Alpha-Terpinolene	11463	Chuanxiong
167	Alpha-Humulene	5281520	Chuanxiong
168	Trans-Beta-Farnesene	5281517	Chuanxiong
169	Ethylisoheptadecanoate	5317250	Chuanxiong
170	Methyl Hexadecanoate	8181	Chuanxiong
171	3-N-Butyl-3-Hydroxy-4,5,6,7-Tetrahydro-6,7-Dihydroxy Phthalide	5315568	Chuanxiong
172	Butylidenecyclohexane	549155	Chuanxiong
173	Methyl Pentadecanoate	23518	Chuanxiong

174	10-(Beta-Hydroxybutyryl)-10-Deacetyl baccatin I	5318160	Chuanxiong
175	Citronellyl Acetate	9017	Chuanxiong
176	Senkyunolide B	5281559	Chuanxiong
177	Ethyl Palmitate	12366	Chuanxiong
178	Chuanxiongzine	14296	Chuanxiong
179	Tetramethylpyrazine	14296	Chuanxiong
180	Methyl Phenylacetate	7559	Chuanxiong
181	Linoleic Acid	5280450	Chuanxiong
182	O-Ethylphenol	6997	Chuanxiong
183	Ethylisooctadecanoate	5317251	Chuanxiong
184	N-Butyl-2-Ethylbutylphthalate	5315567	Chuanxiong
185	Ethyl Hexadecanoate	12366	Chuanxiong
186	Pentadecanoic Acid	13849	Chuanxiong
187	1-Phenyl-1-Pentanone	66093	Chuanxiong
188	2,3-Dihydro-5,7-Dihydroxy-2,6-Dimethyl-8-(3-Methyl-2-Butenyl)-4h-1-Benzopyran-4-One	5316705	Chuanxiong
189	Alpha-Terpinene	7462	Chuanxiong
190	Sabinene	18818	Chuanxiong
191	Cocaine	446220	Chuanxiong
192	Senkyunolide K	5321252	Chuanxiong
193	Humulene	5281520	Chuanxiong
194	Neocnidilide	3083857	Chuanxiong
195	18beta-Glycyrrhetinic Acid	10114	licorice
196	Isoliensinine	5274591	licorice
197	Licoricesaponine C2	6325527	licorice
198	Licorisoflavan A	196831	licorice
199	Isoramanone	5318637	licorice
200	Gancaonin F	5317482	licorice
201	Glycyrol	5320083	licorice
202	Licoricesaponine A3	6325525	licorice
203	Isotrilobine	12310578	licorice
204	Licoricesaponine J2	6325532	licorice
205	Glycyrrhetinic Acid	10114	licorice
206	2,5-Dihydroxymethyl-3,4-Dihydroxypyrrolidine	124702	licorice
207	Gloeosteretriol	132365	licorice
208	Narwedine	10356588	licorice
209	Tetrahydroharmine	159809	licorice
210	2-Methyl-1,3,6-Trihydroxyanthraquinone	5319801	licorice
211	Glycyrin	480787	licorice
212	Neohancoside A	131802	licorice

213	Liquiritigenin-7,4'-Diglucoside	73981649	licorice
214	Gancaonin I	480777	licorice
215	8-Methyl-10-Hydroxylycoctonine	24893580	licorice
216	Ruvoside	101650325	licorice
217	Methylglyoxal	880	licorice
218	Hispaglabridin A	442774	licorice
219	Isoglycyrol	124050	licorice
220	Glycyrrhetol	12310283	licorice
221	Glycyrrhizic Acid	14982	licorice
222	2,4,4'-Trihydroxychalcone	5322052	licorice
223	3'-Methoxyglabridin	5319439	licorice
224	Alpha-Trihydroxy Coprostanic Acid	5322053	licorice
225	Licoricidin	480865	licorice
226	Glisoflavanone	480786	licorice
227	Corylifolinin	5281255	licorice
228	Hispaglabridin B	15228661	licorice
229	Urea	1176	licorice
230	Glyyunnanprosapogenin D	3083297	licorice
231	3-Hydroxyglabrol	480854	licorice
232	Glycyrrhizin	14982	licorice
233	Licobenzofuran	133867	licorice
234	Licoricesaponine G2	11968700	licorice
235	Licoricesaponine D3	11968698	licorice
236	Phaseollinisoflavan	162412	licorice
237	Licoricesaponin C2	452864	licorice
238	Licoricesaponine F3	11968699	licorice
239	Ethyl-N-Buthy-Uralsaponin A Esters	11968460	licorice
240	Methyl-24-Hydroxyglycyrrhetate	5319681	licorice
241	Glyuranolide	195396	licorice
242	5,6,7,8-Tetrahydro-4-Methylquinoline	185667	licorice
243	Gancaonin E	480770	licorice
244	Licocoumarone	503731	licorice
245	Isoliquiritigenin	638278	licorice
246	Neoliquiritin	51666248	licorice
247	Ganoderic Acid A	471002	licorice
248	Sigmoidin B	73205	licorice
249	18alpha-Glycyrrhetinic Acid	73398	licorice
250	Uralsaponin A	128229	licorice
251	Licoricesaponine K2	11968702	licorice
252	Uralsaponin B	163744	licorice
253	Umbelliferone	5281426	licorice
254	Glycycoumarin	5317756	licorice
255	Liquiritin	503737	licorice
256	Liquoric Acid	131751571	licorice

257	Methyl-24-Hydroxy-11-Deoxoglycyrrhetate	5319677	licorice
258	Isotrifoliol	5318679	licorice
259	Liquiritigenin	114829	licorice
260	Glycyrrhisoflavanone	5317762	licorice
261	3-Methyl-6,7,8-Trihydropyrrolo[1,2-A] Pyrimidin-2-One	5319799	licorice
262	Licoleafol	11111496	licorice
263	Tetrahydropalmatine	5417	licorice
264	4'-O-Methylglabridin	5319664	licorice
265	Monoammonium Glycyrrhizinate	62074	licorice
266	Procyanidin B1	11250133	Areca Peel
267	ent-Epicatechin	182232	Areca Peel
